# Informal Coercion Experienced by Adolescents in Mental Health Care—A Systematic Review

**DOI:** 10.1111/inm.70245

**Published:** 2026-03-12

**Authors:** Tiina Överlund, Tella Lantta

**Affiliations:** ^1^ Department of Nursing Science University of Turku Turku Finland; ^2^ Department of Psychiatry Kanta‐Häme Wellbeing County Hämeelinna Finland; ^3^ Centre for Forensic Behavioural Science Swinburne University of Technology Melbourne Australia

**Keywords:** adolescent, experiences, informal coercion, mental health, systematic review

## Abstract

Adolescents are a developmentally vulnerable group in mental health care, yet their experiences of informal coercion remain underexplored. Most existing research reflects adult perspectives, leaving a gap in understanding how adolescents experience such practices. This review synthesises qualitative evidence on the forms and consequences of informal coercion experienced by adolescents in mental health settings. The review followed the PRISMA guidelines and was registered in PROSPERO. A systematic search was conducted across seven databases in February 2025 (PubMed, CINAHL, PsycINFO, EMBASE, Scopus, Web of Science, Cochrane Library). Screening and inclusion were performed using Covidence supplemented by manual reference searches. Qualitative content analysis was applied, using a framework informed by previously identified forms of informal coercion. Across 12 studies, informal coercion shaped adolescents' involvement in mental health care. Predominant forms included treatment pressure, silencing and exclusion and appealing to rules and routines, accompanied by threats or disciplinary control. These practices were experienced as undermining autonomy and trust. The adolescents responded through adaptive and resistant coping strategies, such as compliance, concealment, or overt opposition. The experiences were commonly associated with emotional distress, relational mistrust and hindered recovery, although some adolescents interpreted structured pressure as supportive or protective. Informal coercion is present and consequential in adolescent mental health care. Existing adult‐based conceptualisations may overlook its relational and subtle nature. Further research is needed to explain how informal coercion is constructed in interactions and how it is justified in adolescent mental health care. Such knowledge is essential for developing ethically sound, rights‐respecting nursing practices.

**Trial Registration:** PROSPERO: CRD42025644678

## 1 Introduction

Globally, approximately one in seven adolescents aged 10–19 years experiences a mental health disorder (World Health Organization, Regional Office for Europe 2025), and nearly half of all lifetime mental health conditions begin before the age of 18 (Solmi et al. 2022). The increasing prevalence of mood‐related symptoms has led to a growing demand for adolescent mental health services (Collet et al. 2025). Alongside this, concerns have intensified regarding the quality of care provided to adolescents (World Health Organization, Regional Office for Europe 2025), particularly in relation to the use of coercive practices in mental health treatment (Moell et al. 2024, 2025).

Coercion in mental health care, especially when applied to minors, raises complex ethical questions about legitimacy, efficacy and human rights (Høyer et al. 2002). Evidence suggests that younger patients are disproportionately affected by coercive measures, with age consistently identified as a risk factor (Moell et al. 2024; Walker et al. 2021). This may be linked to developmental vulnerabilities such as limited emotional regulation and consequential thinking, as well as paternalistic attitudes among professionals that lower the threshold for coercion (Moell et al. 2024).

While formal coercion has been widely studied, informal coercion, defined as subtle, non‐legislated pressures used to influence patients' decisions and behaviours, has received considerably less attention (Pelto‐Piri et al. 2019). These practices often remain undocumented and fall outside the scope of clinical guidelines and mental health legislation (Elmer et al. 2018; Yeeles 2016). Previous research on informal coercion has predominantly focused on adult populations and professional perspectives (Andersson et al. 2020; Elmer et al. 2018), with informal coercion conceptualised in diverse ways emphasising its various forms, applications, and broader theoretical foundations (Beeri et al. 2025).

Identified forms of informal coercion include, for example, referring to rules and routines (Pelto‐Piri et al. 2019), deception (Lidz et al. 1998) and treatment pressure (Szmukler and Appelbaum 2008). These practices are often employed to avoid the use of formal coercive measures (Hotzy and Jaeger 2016), to prevent hospitalisation (Yeeles 2016) and to foster treatment engagement (Valenti et al. 2015). In addition to these practical applications, informal coercion has also been discussed as a more abstract phenomenon, frequently described using terms such as ‘softer’ (Allison and Flemming 2019) or ‘subtle’ coercion (Lützén 1998), occupying a conceptual space between full autonomy and formal coercion (Hotzy and Jaeger 2016).

Despite the recognised relevance of informal coercion in psychiatric care (Valenti et al. 2015), evidence regarding its consequences remains limited and inconclusive (Jaeger and Rossler 2010). Moreover, no studies have focused on adolescent patients, despite adolescence being widely recognised as a pivotal developmental phase during which rapid biological, cognitive and social transitions heighten vulnerability to mental health challenges (Fusar‐Poli et al. 2024). The ongoing reliance on formal coercion (Moell et al. 2024; Nyttingnes et al. 2018; Walker et al. 2021), and the possible use of informal coercive practices in adolescent mental health care, highlight a clear research gap, as evidence on their effectiveness and adolescents' lived experiences remains limited. Such research is essential to inform ethically sound and rights‐respecting practices, in line with the mission of the World Psychiatric Association's Child and Adolescent Psychiatry section (2025) (WPA‐CAP), which advocates for the highest standards of clinical and ethical care that uphold the dignity and human rights of adolescent patients.

## 2 Aim

Research on informal coercion in adolescent mental health care is fragmented and conceptually limited. Therefore, this systematic review aimed to synthesise qualitative evidence on informal coercion as experienced by adolescents. The review addresses the following questions:

1. What forms of informal coercion have adolescents experienced during their treatment in mental health care?
2. What are the perceived consequences of informal coercion as experienced by adolescents in mental health care?

## 3 Methods

This systematic review was conducted in accordance with the Preferred Reporting Items for Systematic Reviews and Meta‐Analyses (PRISMA) guidelines (Page et al. 2021) and was registered with PROSPERO, the international register for systematic reviews.

### 3.1 Eligibility Criteria

This systematic review included studies offering qualitative data that allowed adolescents' voices to be authentically represented. Descriptions of the inclusion and exclusion criteria are presented in Table 1. Eligible studies employed qualitative research methods for scientific analysis or mixed‐methods designs that presented relevant qualitative findings. There were no restrictions on publication language or date. In this review, adolescents were defined as individuals aged 13 to 17 years. Studies were included if they focused exclusively on this age group or if the majority of participants (> 50%) were within the 13–17 age range. If the age range of participants was not explicitly reported, studies were included if the described service setting was clearly intended for adolescents. Studies were excluded if they did not involve adolescent participants as defined above, or if they did not focus on adolescents' own first‐hand experiences. Studies conducted outside of mental or psychiatric health care settings were also excluded. No distinction was made between inpatient and outpatient care, as the focus of the review was on the identification of informal coercion in its various forms across different mental health care settings. Thus, there were no restrictions on diagnosis. Furthermore, autobiographical accounts of single individuals that were not subject to scientific analysis were excluded, as were letters, theses, opinion pieces, literature reviews and meta‐analyses that did not present new empirical findings.

**TABLE 1:** Inclusion and exclusion criteria.

	Inclusion	Exclusion
Study Design	Qualitative studies or mixed‐ methods studies including relevant qualitative findings	Quantitative studies or mixed–methods studies not including relevant qualitative findings
Population	Adolescents 13 to 17 years or at least majority (> 50%) of adolescents between 13 and 17 years old.	Study participants were not adolescents as defined in the inclusion criteria
Setting	Inpatient, outpatient, or community mental health care, as defined by each study	Studies conducted outside of mental or psychiatric health care
Phenomena	Adolescents' own lived experiences	Experiences of individuals other than adolescents
Language	Any language	No
Geographic location	Worldwide	No
Publication type	Peer reviewed, empirical research	Auto‐biographical descriptions, letters, theses, opinions and literature reviews or meta‐analyses of existing literature

### 3.2 Search Strategy

A systematic literature search was conducted in February 2025 across seven electronic databases: PubMed, CINAHL, PsycINFO, EMBASE, the Cochrane Library, Scopus and Web of Science. In addition to database searches, reference lists of the included studies were screened for potentially relevant articles, and manual searches were performed using Google Scholar to identify additional eligible studies. The selection of key words was informed by pilot searches, including studies focusing on informal coercion across various population groups and mental health contexts, and the final search strategy was externally validated in consultation with a specialist university librarian. To structure the search, the PICO model was applied, focusing on population (adolescents), intervention (informal coercion) and outcome (experiences). Search terms included broad combinations of terms, including (i) adolescent, (ii) mental health or psychiatric care, (iii) informal coercion and other terms referring to informal coercion based on previous literature. The complete search strategies for each database are provided in Table S1.

### 3.3 Study Selection

The screening of studies was conducted independently by two blinded reviewers (T.Ö., S.B.) in Covidence. During the screening process, a few discrepancies in eligibility assessments arose. These were discussed among the reviewers, and the senior author (T.L.) was consulted to reach a consensus.

### 3.4 Quality Appraisal

The quality of the included studies was assessed using the Mixed Methods Appraisal Tool (MMAT), which enables the evaluation of quantitative, qualitative and mixed‐methods studies with a single instrument. Each study was appraised using seven criteria, with response options of ‘Yes’, ‘No’, or ‘Can't tell’ (Hong et al. 2019). Two reviewers (T.Ö., S.B.) conducted the assessments independently. In cases of disagreement, the senior author (T.L.) reviewed the assessments and made the final decision.

### 3.5 Data Extraction

Data extraction was conducted independently and manually by two authors (T.Ö., S.B.). Any discrepancies were resolved through discussion, and unresolved cases were adjudicated by the senior author (T.L.). General study characteristics were extracted, including authorship, publication year and country of origin. Data were also collected on the study aim, methodological design and participant characteristics, such as the total sample size, mental health care setting and diagnostic information.

Data extraction related to informal coercion was conducted by two reviewers (T.Ö., S.B.), guided by a deductive framework developed based on prior empirical and theoretical literature (T.Ö.). The framework outlined previously recognised forms of informal coercion with corresponding operational definitions (Lidz et al. 1998; Lorem et al. 2015; Neale and Rosenheck 2000; Pelto‐Piri et al. 2019; Potthoff et al. 2022; Rugkåsa et al. 2014; Szmukler and Appelbaum 2008; Valenti et al. 2015), and was systematically applied across all included studies to enable structured mapping of these forms. The complete framework and operational definitions are presented in Table S2.

### 3.6 Data Analysis

Data were analysed using deductive (Elo and Kyngäs 2008) and inductive (Hsieh and Shannon 2005) qualitative content analysis. Deductive analysis was conducted first, in which predefined categories from the framework were used to code and organise the extracted data. Consensus decisions were reached through discussion between the reviewers (T.Ö., S.B.), and final arbitration was provided by the senior author (T.L.). Forms of informal coercion that did not align with the predefined categories but were identified as relevant were analysed using inductive content analysis by (T.Ö.). These emergent expressions were reviewed collaboratively (T.Ö., S.B.), and consensus was achieved through discussion. Disagreements were resolved by consulting the senior author (T.L.).

To gain an understanding of the magnitude of different forms of informal coercion in adolescents' experiences, the identified expressions were quantified by counting their occurrences across the included studies (T.Ö.). This quantitative mapping enabled a descriptive representation of the relative prevalence of each form of informal coercion.

## 4 Results

### 4.1 Study Selection Results

**FIGURE 1:**
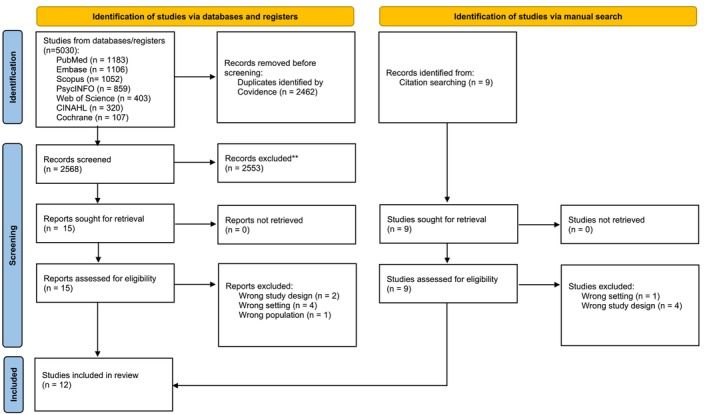
PRISMA flow diagram of the study selection process.

A total of 5039 records were identified through electronic database searches (*n* = 5030) and manual citation tracking (*n* = 9). After removing duplicates (*n* = 2462), 2568 records remained for title and abstract screening, of which 2553 were excluded. In the full‐text screening phase, 15 articles were assessed for eligibility, resulting in the exclusion of seven articles. Ultimately, 12 (*n* = 12) studies met the inclusion criteria and were included in the review (Andrea Gonzalez‐Urbina 2022; Bjønness et al. 2020; Coyne et al. 2015; Engström et al. 2020; Jones et al. 2021; LeFrançois 2008; Ludot‐Grégoire et al. 2022; Moses 2011; Offord et al. 2006; Persson et al. 2017; Rice et al. 2021; Tan et al. 2010). The screening process is presented in the flow chart below (Figure 1).

### 4.2 Study Characteristics

The study characteristics from the included studies are presented in Table 2. Three of the studies were conducted in the United Kingdom (LeFrançois 2008; Offord et al. 2006; Tan et al. 2010), three in the United States (Jones et al. 2021; Moses 2011; Rice et al. 2021) and two in Sweden (Engström et al. 2020; Persson et al. 2017). One study was identified from each of the following countries: Ireland (Coyne et al. 2015), France (Ludot‐Grégoire et al. 2022) and Norway (Bjønness et al. 2020). Only one study was conducted in a non‐Western country, Chile (Andrea Gonzalez‐Urbina 2022).

**TABLE 2:** Study characteristics.

Study, author (years), country	Design	Aim	Patient description (total sample (*n*), sex ratio (*n*/%), age (mean), diagnosis, treatment setting)	Informal coercion		Main results
				Informal coercion forms	Consequences	
Bjønness et al. (2020), Norway	Exploratory qualitative design	To explore adolescents' experiences with user participation and shared decision‐making in Child and Adolescents Mental Health Services (CAMHS) inpatient units	*n* = 10 Age: 16–18 years (mean 16.5) Sex: Female (*n* = 8, 80%), Male (*n* = 2, 20%) Dg: Self‐reported diagnosis (*n* = 9); Autism spectrum disorder (*n* = 2), Anxiety (*n* = 5), psychosis (*n* = 2), Depression (*n* = 5), ADHD (*n* = 3), Eating disorder (*n* = 1), Trauma/PTSD (*n* = 1) Treatment setting: Child and adolescent psychiatric inpatient units (CAMHS)	Treatment pressure, silencing and exclusion, deception	Reactive coping strategies, emotional wellbeing, relational impacts on the care relationship	Experiences of social pressure to adhere to care, engage in therapy and meet others' expectations. Intentional restriction of participation through exclusion. Deceptive referral and misleading therapeutic framing. Impacts led to resistance, aggression, disappointment, tactical adaptation and submission
Coyne et al. (2015), Ireland	Descriptive qualitative design	To explore adolescents' and parents' experiences of CAMHS in relation to accessibility, approachability and appropriateness	*n* = 15 Age: 11–17 years, mean N/A Sex: Female (*n* = 9, 60%), Male (*n* = 6, 40%) Dg: Mood disorder (*n* = 5), Attention deficit hyperactivity disorder (*n* = 3), impulse control, anxiety, adjustment and behavioural disorders (*n* = 7) Treatment setting: Community CAMHS outpatient clinics for children and adolescents	Silencing and exclusion, treatment pressure	N/A	Experiences of deliberate restriction of participation by excluding the individual from decision‐making related to their own care. Social pressure to meet externally imposed expectations regarding commitment to treatment
Engström et al. (2020), Sweden	Descriptive qualitative design	To explore adolescents' views on how staff relate and perform their duties, favourable characteristics among staff, consequences of different treatment from staff and their safety experiences	*n* = 20 Age: 13–19 years (mean N/A) Sex: Female (*n* = 13, 65%), male (*n* = 7, 35%) Dg: N/A Treatment setting: Coercive youth care institutions and psychiatric inpatient settings for compulsory care	Threat, using a disciplinary style, referring to rules and routines, physical force	Emotional wellbeing, relational impacts on the care relationship	Adolescents reported escalating forms of coercion, including verbal threats, sanctions and punishments to enforce compliance. Unit rules were experienced both as care and as instruments of control. Staff were described as using physical pressure, violence and humiliating behaviour
Gonzalez‐Urbina (2022), Chile	Descriptive qualitative study	To understand the experience of C&As on coercive practices during their stay in a psychiatric ward	*n* = 14 Age: 9–15 years (mean N/A) Sex: N/A Dg: N/A Treatment setting: Closed child and adolescent psychiatric inpatient ward using coercive practices	Threat, using a disciplinary style, referring to rules and routines	Emotional wellbeing, relational impacts on the care relationship	Experiences of behavioural control through threats. Disciplinary regulation through collective punishment and sanctions. Inhumane rules that disrupted individuality and connection to other individuals. These practices caused negative emotional impacts, including feelings of worthlessness and hopelessness
Jones et al. (2021), United States	Grounded theory	To explore how initial involuntary hospitalisations impact youth and young adult treatment pathways following discharge	*n* = 40 Age: During treatment 11–23 years (mean16.2), during interviews 16–27 years; mean (19.4) Sex: Female *n* = 28 (70%), Gender‐fluid or non‐binary *n* = 1 (2.22%) Dg: N/A Treatment setting: Psychiatric involuntary inpatient unit	Silencing and exclusion, threat	Reactive coping strategies	Experiences of restricted participation through exclusion from treatment‐related decision‐making, coercion into medical examinations without involvement or influence, and threats of more coercive treatment (e.g., involuntary care). These practices led to behavioural responses such as concealment of true wellbeing, deliberate silence and feigning improved condition
LeFrançois (2008), United Kingdom	Ethnographic research	To observe and document the ways in which children's participation rights are implemented within an adolescent psychiatric inpatient unit	*n* = N/A (inpatient ward with patient beds for 8 to 10) Age: 11–18 years (mean N/A) Sex: N/A Dg: N/A Treatment setting: Secure child and adolescent psychiatric inpatient unit	Silencing and exclusion, threat, using a disciplinary style, treatment pressure	Emotional wellbeing	Deliberate exclusion from treatment decisions and disregard of adolescents' views. No options or choices were offered. Informal coercion was implemented through threats of punishment and sanctions for non‐compliance. Social pressure to exceed personal boundaries in therapy. Resulted in emotions of worthlessness
Ludot‐Grégoire et al. (2022), France	Qualitative study with interpretative phenomenological analysis	To explore the perspectives of adolescents with anorexia nervosa on antidepressants	*n* = 15 Age: 12–25 years (mean 17.4 years) Sex: Female (*n* = 13, 87%), Male (*n* = 3, 13%) Dg: Anorexia Nervosa (*n* = 15) Treatment setting: Child and adolescent psychiatric inpatient and outpatient unit for eating disorders (hospital‐based CAMHS)	Silencing and exclusion	Reactive coping strategies	Experiences of medication treatment without opportunities for influence. Exclusion and restricted participation led to tactical adaptation, including one‐sided negotiation attempts and reluctant compliance
Moses (2011), United States	Mixed‐methods	To examine adolescents' commitment to continuing psychotropic medication in the absence of external pressure, and to identify the subjective experiences and individual, clinical, social and demographic factors associated with greater or lesser commitment	*n* = 60 Age: 12–17 years (mean 14.8) Sex: Male (*n* = 29, 58%) Dg: ADD/HD (*n* = 29), Depression (*n* = 18), Bipolar disorder: (*n* = 16), Conduct disorder (*n* = 14); Anxiety (including OCD) (*n* = 12), Oppositional defiance disorder (ODD) (*n* = 11), PTSD (*n* = 9), Mood disorder (*n* = 8), Reactive attachment disorder (*n* = 7), Alcohol or drug abuse or dependence (*n* = 6) Treatment setting: Intensive community‐based wraparound mental health services (outpatient)	Silencing and exclusion, threat, physical force	Emotional wellbeing, reactive coping strategies, impacts on health behaviour	Experiences of treatment effectiveness were consciously disregarded. Medication adherence was enforced through threats of punishment and physical coercion. These practices led to feelings of hopelessness, treatment discontinuation as resistance, and occasionally increased motivation for care
Offord et al. (2006), United Kingdom	Descriptive qualitative design with interpretative phenomenological analysis	To explore young adults' views regarding: the inpatient treatment they received for anorexia nervosa during their adolescences; their experiences of discharge; and the impact their admission had on issues of control and low self‐esteem	*n* = 7 Age: 16–23 years (mean N/A) Sex: Female (*n* = 7, 100%) Dg: Anorexia Nervosa (*n* = 7) Treatment setting: General adolescent psychiatric inpatient units providing treatment for anorexia nervosa	Silencing and exclusion, manipulation, referring to rules and routines	Relational impacts on the care relationship, reactive coping strategies	Experiences of stigmatisation used to justify treatment and control extending beyond therapeutic goals. The young person was disregarded, and individual needs were consciously overlooked. These practices led to resistance, loss of trust in professionals and dishonest behaviour
Persson et al. (2017), Sweden	Explorative qualitative design	To investigate young service users' views of outpatient and community mental health clinics in Sweden, based on two data sources	Phase I (Focus groups): *n* = 6 Age: 10–18 years (mean N/A), Sex: Focus groups: Female (*n* = 3, 50%); Male (*n* = 3, 50) Dg: N/A Treatment setting: Outpatient and community‐based child and adolescent mental health services (CAMHS)	Treatment pressure	N/A	Adolescents described pressure to engage in treatment from their social environment. During clinical encounters, adolescents reported feeling pressured to say the ‘right’ things and to overstep their own boundaries by disclosing more than they wished
Rice et al. (2021), United States	Explorative qualitative design	To understand how youth navigated the course of their treatment from the time of admission to the time of discharge	*n* = 25 Age: 13–17 years (mean 15.8) Sex: Female (*n* = 15, 60%), Male (*n* = 8, 32%), Transgender Female (*n* = 1, 4%), Transgender Male (*n* = 1, 4%) Dg: Major Depressive Disorder (MDD) (*n* = 25), Eating disorder (*n* = 1), Cannabis Abuse (*n* = 2), Opioid Abuse (*n* = 1), ADHD (*n* = 3), Oppositional Defiant Disorder (ODD) (*n* = 6), PTSD (*n* = 1) Treatment setting: Acute involuntary psychiatric inpatient unit for adolescents and young adults	Manipulate, referring to rules and routines	Emotional wellbeing, impacts on recovery	Standardised ward practices, such as the removal of personal belongings, were perceived as dehumanising and prison‐like, reinforcing adolescents' feelings of marginalisation and lack of participation. While these measures enhanced safety, they also reinforced feelings of marginalisation and hindered recovery
Tan et al. (2010), United Kingdom	Descriptive qualitative design	To explore the views of 29 young women concerning compulsion and coercion in the treatment of anorexia nervosa	*n* = 29 Age: 15–16 years (median 17, mean 18) Sex: Female (*n* = 29, 100%) Dg: Anorexsia Nervosa (*n* = 29) Treatment setting: Child and adolescent psychiatric inpatient units for compulsory treatment of anorexia nervosa	Threat	Impacts on recovery, relational impacts on the care relationship, emotional wellbeing	Experiences of being threatened with more coercive or involuntary treatment in order to accept voluntary care. These practices were perceived both as expressions of care, and conversely as ineffective, hindering recovery and exacerbating symptoms. Such experiences contributed to conflict and mistrust in the therapeutic relationship. Participants reported feeling powerless, disappointed and angry

Six (*n* = 6) of the studies were conducted in the 2020s (Gonzalez‐Urbina 2022; Bjønness et al. 2020; Engström et al. 2020; Jones et al. 2021; Ludot‐Grégoire et al. 2022; Rice et al. 2021), four (*n* = 4) in the 2010s (Coyne et al. 2015; Moses 2011; Persson et al. 2017; Tan et al. 2010) and two (*n* = 2) in the 2000s (LeFrançois 2008; Offord et al. 2006). Two (*n* = 2) of the studies were retrospective, based on participants' recollections of past treatment experiences occurring 1 month to 6 years (Jones et al. 2021) and 2 to 5 years (Offord et al. 2006) prior to data collection.

The included studies were conducted across diverse child and adolescent mental health care settings, encompassing both inpatient and outpatient contexts. Several studies were situated in inpatient psychiatric units, including acute, long‐term and compulsory care wards as well as specialised settings such as intensive care and crisis stabilisation units (Bjønness et al. 2020; Engström et al. 2020; Gonzalez‐Urbina 2022; Rice et al. 2021; Tan et al. 2010). Others were conducted in multidisciplinary hospital departments or specialised programmes providing adolescent mental health care, such as day hospitals or inpatient eating disorder units (Ludot‐Grégoire et al. 2022; Offord et al. 2006). A number of studies were conducted in outpatient and community‐based services, including CAMHS clinics and wraparound programmes offering psychosocial and therapeutic support (Coyne et al. 2015; Moses 2011; Persson et al. 2017). In addition, some studies were conducted in integrated or mixed‐care settings providing inpatient, day‐patient and community services (LeFrançois 2008; Jones et al. 2021).

Across the reviewed studies, a total of 242 participants were included, with ages ranging from 9 to 26 years. Where reported, mean participant ages varied between 14.8 and 18.1 years (Bjønness et al. 2020; Jones et al. 2021; Rice et al. 2021; Tan et al. 2010). While several studies encompassed broad age ranges extending into late adolescence and early adulthood, the majority focused more narrowly on mid‐adolescent populations. Sample sizes ranged from 7 to 60 participants. One study did not report the number of participants but described the inpatient setting as providing beds for 8 to 10 children (LeFrançois 2008). Among the studies that reported participants' gender, approximately 70% included samples in which females constituted the majority.

The data on adolescents' family structures and living environments varied notably across the reviewed studies. In one study, 54% of the participants were reported to live outside the parental home, in foster care, residential facilities or group homes (Moses 2011). Another two studies referred more generally to some participants being from foster care institutions, experiencing unstable home environments (Jones et al. 2021; Rice et al. 2021), or being homeless (Jones et al. 2021). The remaining studies did not specify the living environment or family conditions of the adolescents.

Slightly more than half of the studies (*n* = 7) provided detailed diagnostic information about the adolescents. In addition, broader descriptions of symptoms were reported, including internalising and externalising symptoms as well as family‐related causes (Persson et al. 2017), or more generally as severe mental health problems (LeFrançois 2008), indicating a wide spectrum of mental health conditions among participants. The most frequently reported diagnostic category was mood disorders, including depression and anxiety, either as primary diagnoses or comorbid conditions, affecting a total of 93 adolescents (Bjønness et al. 2020; Coyne et al. 2015; Ludot‐Grégoire et al. 2022; Moses 2011; Rice et al. 2021). The second most common diagnostic group was eating disorders, particularly anorexia nervosa, reported in 53 adolescents (Bjønness et al. 2020; Ludot‐Grégoire et al. 2022; Offord et al. 2006; Rice et al. 2021; Tan et al. 2010). Furthermore, neurodevelopmental disorders emerged as the third most frequently reported diagnostic category, affecting 38 adolescents (Bjønness et al. 2020; Coyne et al. 2015; Rice et al. 2021).

### 4.3 Quality of Included Studies

Of the included studies, 11 employed qualitative methodologies, and one was a mixed‐methods study with a qualitative component. Table 3 summarises the quality assessment results. All included studies received quality assessment scores of 7 out of 7. The mixed‐methods study was rated 13 out of a possible 17 points according to the relevant appraisal criteria. The included studies were predominantly conducted in Western countries, with only one study originating from a non‐Western setting (Gonzalez‐Urbina 2022). Such geographical bias may restrict the applicability and generalisability of the findings to culturally diverse populations. Furthermore, male underrepresentation was notable in two studies that did not include any male participants (Offord et al. 2006; Tan et al. 2010); consequently, the findings may not comprehensively reflect the broader adolescent population.

**TABLE 3:** Quality appraisal of the included studies using the MMAT.

References	Screening questions		Qualitative studies					Quantitative non‐randomised studies					Mixed‐methods studies					Total points
Andrea Gonzalez‐Urbina (2022)	Y	Y	Y	Y	Y	Y	Y											7/7
Bjønness et al. (2020)	Y	Y	Y	Y	Y	Y	Y											7/7
Coyne et al. (2015)	Y	Y	Y	Y	Y	Y	Y											7/7
Engström et al. (2020)	Y	Y	Y	Y	Y	Y	Y											7/7
Jones et al. (2021)	Y	Y	Y	Y	Y	Y	Y											7/7
LeFrançois (2008)	Y	Y	Y	Y	Y	Y	Y											7/7
Ludot‐Grégoire et al. (2022)	Y	Y	Y	Y	Y	Y	Y											7/7
Moses (2011)	Y	Y	Y	Y	Y	Y	Y	N	Y	Y	CT	N/A	Y	Y	Y	CT	Y	13/17
Offord et al. (2006)	Y	Y	Y	Y	Y	Y	Y											7/7
Persson et al. (2017)	Y	Y	Y	Y	Y	Y	Y											7/7
Rice et al. (2021)	Y	Y	Y	Y	Y	Y	Y											7/7
Tan et al. (2010)	Y	Y	Y	Y	Y	Y	Y											7/7

Abbreviations: CT, can't tell based on the article; N, no; Y, yes.

The studies were conducted across a wide range of mental health service settings, allowing for a broad exploration of informal coercion in diverse contexts. However, the descriptions of service environments were not consistently detailed (Ludot‐Grégoire et al. 2022). Moreover, none of the studies explicitly addressed informal coercion as their primary research focus, which underscores the contextual and experiential nature of the data from adolescents' perspectives. While the interpretation of findings appeared to be well‐supported across the studies, the contextual dependency of adolescents' experiences could have been articulated more explicitly (Jones et al. 2021; Offord et al. 2006; Persson et al. 2017).

While ethical considerations were generally well addressed and the studies were well justified, reflexivity remained largely methodological and did not extend to a critical examination of the researchers' roles. It is worth noting that only two of the included studies explicitly addressed reflexivity (Gonzalez‐Urbina 2022; Jones et al. 2021). In addition, two studies reported the involvement of researchers with personal experience (Jones et al. 2021) or youth co‐researchers (Bjønness et al. 2020) in the research process. These participatory elements may enhance the credibility and relevance of the findings, although their limited presence across the studies suggests that youth perspectives were not consistently integrated into the research design or interpretation.

### 4.4 Informal Coercion Forms Experienced by Adolescents

From the data included in this review, seven of the 13 previously distinct forms of informal coercion were identified: interpersonal leverage, threat, (using) a disciplinary style, referring to rules and routines, treatment pressure, deception and physical force. Furthermore, the analysis of adolescents' experiences revealed two additional distinct forms of informal coercion: silencing and exclusion and manipulation. Table 4 presents the forms of informal coercion included in the deductive framework developed from previous research and those emerging inductively from this systematic review. The numbers (*n*) refer to the total number of coded expressions representing each form of informal coercion across all included studies.

**TABLE 4:** Number of informal coercion forms identified in the current systematic review and their relationship to previous research (total *n* = 69).

Informal coercion		
Relationship to previous research	Form of informal coercion	Number of identified expressions
Previously identified forms, deductively identified in the current review	Treatment pressure	*n* = 17
	Referring to rules and routines	*n* = 12
	Threat	*n* = 10
	(Using) a disciplinary style	*n* = 7
	Physical force	*n* = 5
	Deception	*n* = 1
	Interpersonal leverage	*n* = 1
Previously identified forms not identified in the current review	Persuasion	Not identified
	Inducement	Not identified
	Influencing behaviour	Not identified
	Blackmail	Not identified
	Show of force	Not identified
	Giving orders	Not identified
Forms emerging inductively from the current review	Silencing and exclusion	*n* = 14
	Manipulation	*n* = 2

Abbreviations: *n* = number of identified expressions for each form of informal coercion; Not identified = no expressions identified in the included studies.

**FIGURE 2:**
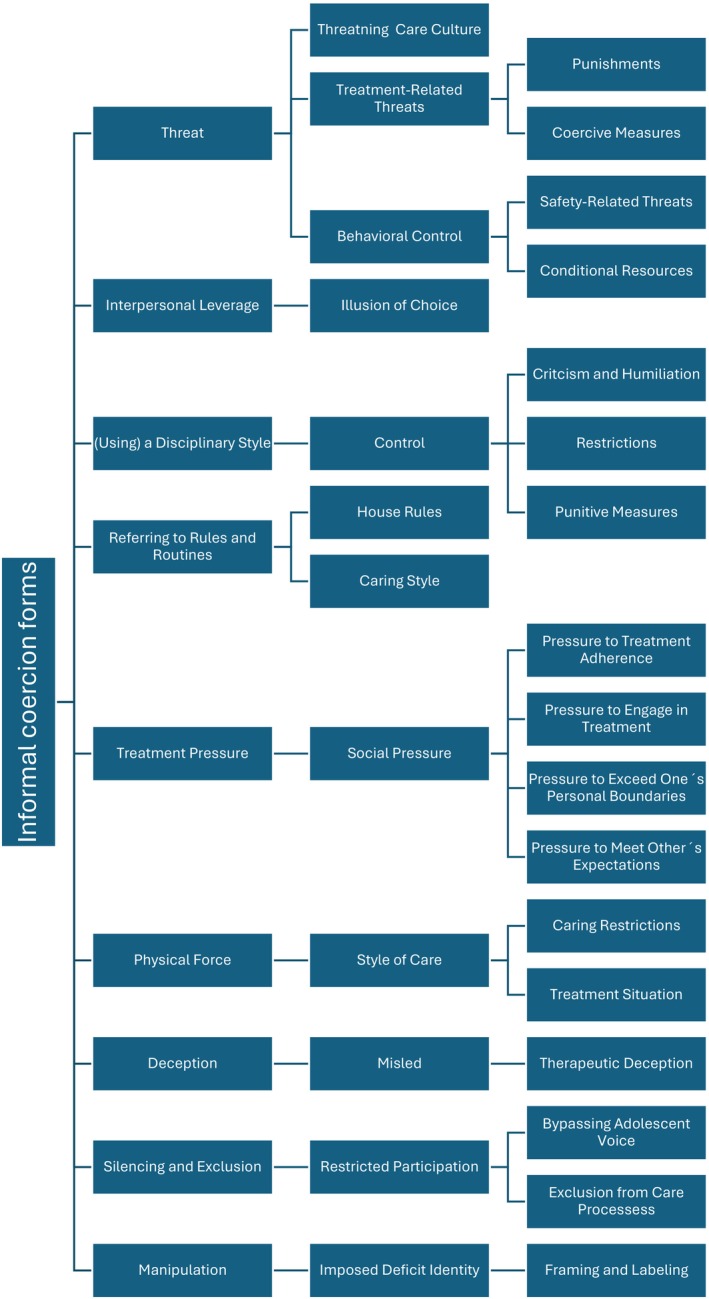
Forms of informal coercion identified in the included studies.

In total, 69 (*n* = 69) expressions reflecting adolescents' experiences of informal coercion were identified across the included studies. Among adolescents, the most frequently reported forms of informal coercion were treatment pressure (*n* = 17), silencing and exclusion (*n* = 14), and referring to rules and routines (*n* = 12). These were followed by experiences of threats (*n* = 10) and using a disciplinary style (*n* = 7). Notably, some adolescents reported experiencing physical force (*n* = 5) not associated with formal coercive measures such as restraints. Less frequently, adolescents described experiences of manipulation (*n* = 2), interpersonal leverage (*n* = 1) and deception (*n* = 1). An overview of the results of experienced informal coercion forms is presented in Figure 2.

#### 4.4.1 Threat

In the reviewed studies, Threat (*n* = 10) emerged in adolescents' experiences as an action manifested through threatening care culture, treatment‐related threats, or threats aimed at controlling the behaviour of adolescents. Threats were perceived as a method of care and, in some cases, as part of a professional's personal style of practice, often characterised by a threatening communication style and authoritarian behaviour (Engström et al. 2020). Adolescents reported being threatened with involuntary treatment or more coercive treatment measures to ensure compliance with care initiation and behavioural regulation within care units (Engström et al. 2020; Jones et al. 2021; Tan et al. 2010). For example, coercive threats were used to persuade adolescents to consent to treatment (Tan et al. 2010), while punishing actions, such as removal of privileges (Moses 2011) or denial of discharge or weekend leave (LeFrançois 2008), were employed to maintain engagement. In addition to threats of coercion, adolescents' behaviour was shaped through conditional access to personal resources, such as their belongings (Gonzalez‐Urbina 2022). Safety‐related threats, including threatening to activate alarm systems in response to unwanted behaviour, were also used to influence adolescents' actions (Engström et al. 2020). In reviewed studies, these threatening practices were described by adolescents as instruments of power, strategically employed to enforce compliance and exert control over adolescents' conduct.

#### 4.4.2 Interpersonal Leverage

One expression (*n* = 1) was categorised under interpersonal leverage. Pressure and feelings of guilt directed toward family members and professionals created only an illusion of freedom of choice. Although the decision to initiate treatment was technically perceived as self‐made, it was influenced by a sense of guilt toward loved ones and a desire to please others (Tan et al. 2010).

#### 4.4.3 (Using) a Disciplinary Style

In the reviewed studies, using a disciplinary style (*n* = 7) was identified as a means of control, manifested through criticism and humiliation, restrictions and punitive measures, such as collective sanctions. Professionals sought to influence adolescents' behaviour and presence using disciplinary methods, which included collective punishment, public criticism and humiliation of individual youths (Gonzalez‐Urbina 2022). Treatment plans were described as disciplinary tools, with obedience expected from the adolescents (Engström et al. 2020). Participants reported that it was made clear that failure to comply with the treatment plan would result in disciplinary actions, such as emergency meetings or punitive consequences (LeFrançois 2008).

#### 4.4.4 Referring to Rules and Routines

Expressions categorised under referring to rules and routines (*n* = 12) captured adolescents' experiences of both the prevailing style of care adopted by professionals and the house rules that structured daily life. House rules often referred to generalised routines that embodied impersonal, dehumanising and non‐individualised practices, reflecting a care environment detached from adolescents' personal needs (Engström et al. 2020; Offord et al. 2006; Rice et al. 2021). House rules were perceived as guiding treatment without regard for individual circumstances (Andrea Gonzalez‐Urbina 2022; Offord et al. 2006). They were broadly applied to restrict freedom of movement (Gonzalez‐Urbina 2022), communication (Rice et al. 2021) and peer interaction (Gonzalez‐Urbina 2022; Rice et al. 2021), and were often described as excessive, arbitrary and unjust (Engström et al. 2020; Offord et al. 2006). Beyond their regulatory function, rules and routines were experienced as a style of care characterised by a rule‐oriented and coercive approach, and manifested in interactions with adolescents that lacked therapeutic justification (Engström et al. 2020; Offord et al. 2006). At times, they were used to influence behaviour without explanation and functioned as instruments of coercion, with compliance expected as a sign of respect (Engström et al. 2020).

#### 4.4.5 Treatment Pressure

In the reviewed studies, Treatment Pressure (*n* = 17) was experienced as a form of social pressure. This category was further divided into pressure to meet others' expectations, pressure to adhere to treatment, pressure to engage in treatment and pressure to exceed one's personal boundaries during the treatment process. Adolescents reported being pressured to comply with decisions made by others (Bjønness et al. 2020) or to participate in treatment activities, such as group sessions, according to externally imposed performance expectations (LeFrançois 2008). In treatment meetings, they felt compelled to say the ‘right’ things and to perform within predefined boundaries, including pressure to begin therapeutic work according to professionals' timelines (Persson et al. 2017). Pressure to engage in treatment was experienced from professionals, family members and peers on the ward, even when the adolescent lacked personal motivation or readiness (Bjønness et al. 2020; Persson et al. 2017). Adolescents who acted differently were required to justify their choices, which was sometimes deemed insufficient (LeFrançois 2008). They described feeling pressured to accept ‘voluntary’ treatment, in which their opinions were solicited in ways that did not allow for refusal (Coyne et al. 2015; Tan et al. 2010). Additionally, they experienced pressure to exceed personal boundaries, such as disclosing more than they were comfortable with or participating in interventions they found unpleasant or unhelpful (Persson et al. 2017).

#### 4.4.6 Physical Force

In reviewed studies Physical Force (*n* = 5) was identified as a style of care within the caring relationship or as a specific treatment situation. Adolescents described interactions with professionals in which, despite the absence of formal coercion, they were physically pressured or compelled to act in a prescribed manner. Physical Force was experienced as humiliating and, at times, as physical violence used to ensure obedience (Engström et al. 2020). Such experiences occurred both within professional–adolescent interactions (Engström et al. 2020) and in family contexts, in which a parent was reported to have physically coerced the adolescent into taking medication (Moses 2011).

#### 4.4.7 Deception

Deception (*n* = 1) captured adolescents' experiences of being deceived into treatment without having any genuine influence over the decision. Adolescents described feeling misled into care, reflecting a lack of transparency and autonomy in the admission process (Bjønness et al. 2020).

#### 4.4.8 Silencing and Exclusion

Across the reviewed studies, Silencing and Exclusion (*n* = 14) was identified as a form of informal coercion that reflected a restriction of participation, which referred to the exclusion from care processes and bypassing adolescents' voices, particularly in decisions related to their care. Such exclusion was often enacted through professionals' intentional communication strategies and practices that bypassed adolescents in treatment‐related decision‐making (Bjønness et al. 2020; Coyne et al. 2015; LeFrançois 2008; Moses 2011). Adolescents' perspectives were frequently disregarded, especially in decisions concerning treatment plans or interventions (Coyne et al. 2015; LeFrançois 2008; Ludot‐Grégoire et al. 2022; Moses 2011). In some cases, treatments or actions related to care were administered without clear justification or without offering the adolescent any meaningful choice (Jones et al. 2021; Offord et al. 2006). Requests to review medication (LeFrançois 2008; Moses 2011) or to engage in treatment planning (Coyne et al. 2015; LeFrançois 2008) were routinely overlooked, and opportunities to express preferences regarding personally meaningful treatment options were notably absent (Moses 2011).

#### 4.4.9 Manipulation

In the reviewed studies, Manipulation (*n* = 2) was identified as a form of informal coercion, experienced through framing and labelling. It shaped adolescents' experiences of care through professionals' assumptions, interpretations and directive practices (Offord et al. 2006; Rice et al. 2021). Professionals were perceived as treating adolescents according to diagnostic stereotypes, such as assuming dishonesty in individuals with eating disorders, which led to the rejection of their narratives and a loss of personal agency (Offord et al. 2006). Furthermore, adolescents reported that the structure and practices of the treatment environment reinforced the belief that there was something fundamentally wrong with them. Adolescents described the institutional routines as so dehumanising that they began to believe there must be something inherently wrong with them, thus legitimising the need for treatment. The framing of their thoughts and behaviours as symptoms served to justify treatment without further explanation or dialogue (Rice et al. 2021). This deficit‐based identity was not self‐derived but constructed through institutional routines and interactions, which shaped how adolescents came to understand themselves within the context of care.

### 4.5 Adolescents' Experienced Consequences

**FIGURE 3:**
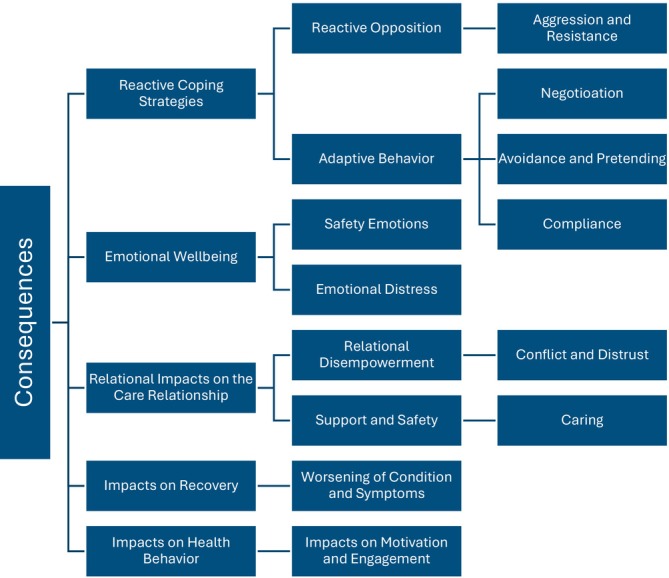
Consequences of informal coercion identified in the included studies.

This review also aimed to explore the consequences of informal coercion experienced by adolescents. Attention was directed toward identifying the specific consequences informal coercion has on different dimensions of experience. A total of 41 expressions (*n* = 41) of consequences were identified in the data that could be linked to experiences of informal coercion. Based on the content of these expressions, five main categories were constructed: Reactive coping strategies (*n* = 15), emotional wellbeing (*n* = 13), relational impacts on the care relationship (*n* = 9), impacts on recovery (*n* = 3) and impacts on health behaviour (*n* = 1). The consequences of informal coercion are outlined below and illustrated in more detail in Figure 3.

#### 4.5.1 Reactive Coping Strategies

Adolescents' responses to informal coercion were described as reactive coping strategies (*n* = 15), manifesting either as adaptive behaviour or reactive opposition. Adaptive behaviour included acts of compliance, negotiation, avoidance and pretending, such as deliberate lying, feigning wellness, withholding information and attempting to influence treatment decisions through interaction with professionals (Bjønness et al. 2020; Jones et al. 2021; Ludot‐Grégoire et al. 2022; Offord et al. 2006). In contrast, reactive opposition was expressed through acts of resistance and aggression, including non‐adherence to medication, disengagement from treatment plans (Moses 2011) and overt behaviours such as shouting or throwing objects, often triggered by feelings of exclusion and lack of agency (Bjønness et al. 2020). These strategies were not driven by intrinsic motivation but rather by a perceived lack of alternatives (Bjønness et al. 2020; Ludot‐Grégoire et al. 2022; Moses 2011). In some cases, resistance was described as the only available means of exerting influence (Bjønness et al. 2020). Importantly, opposition was not limited to individual professionals; it was also directed at broader service structures or punitive institutional practices (Offord et al. 2006).

#### 4.5.2 Emotional Wellbeing

The consequences on Emotional Wellbeing (*n* = 13) identified in the reviewed studies were grounded in individual affective experiences of adolescents, which were attributed to the influence of informal coercion. These experiences were classified into emotional distress perceived as negative consequences and positive emotional responses. Notably, none of the included studies reported ambivalent expressions. Adolescents frequently described feelings of powerlessness, sadness and anger, which were attributed to the limited opportunities for influence and autonomy (Engström et al. 2020; Moses 2011; Offord et al. 2006). Experiences of worthlessness, disappointment and frustration were commonly reported, particularly in interactions with professionals perceived as rigid in their adherence to rules, routines or disciplinary practices (Engström et al. 2020).

Furthermore, adolescents described emotional responses of bitterness resulting from being ignored, belittled or treated in a punitive manner (Engström et al. 2020; Tan et al. 2010). Collective punishments were associated with feelings of hopelessness (Gonzalez‐Urbina 2022). Withholding treatment options from adolescents and the experience of being coerced without formal justification were perceived as mentally burdensome and, in some cases, likened to psychological torture (Bjønness et al. 2020; LeFrançois 2008). Despite the predominance of negative emotional consequences, one study also highlighted positive emotional experiences. These were primarily associated with safe and consistent structures that enabled adolescents to focus on themselves and their treatment, while simultaneously preventing self‐harming behaviours (Rice et al. 2021).

#### 4.5.3 Relational Impacts on the Care Relationship

Informal coercion was identified as influencing care relationships (*n* = 9), particularly through two relational dimensions: relational disempowerment and experiences of support and safety. In several studies (Gonzalez‐Urbina 2022; Bjønness et al. 2020; Engström et al. 2020; Offord et al. 2006; Tan et al. 2010), adolescents were described in ways suggesting a subordinate and powerless position within care settings, often characterised by conflict and mistrust. A reliance on rules and routines was interpreted as undermining respect and trust toward professionals (Engström et al. 2020), while restricting participation and choices appeared to reinforce adolescents' sense of disempowerment and mistrust (Moses 2011; Offord et al. 2006; Tan et al. 2010). Practices such as collective punishment were perceived by adolescents as oppressive, evoking prison‐like experiences (Gonzalez‐Urbina 2022; Bjønness et al. 2020). In contrast, some expressions reflected a more positive relational dynamic, captured under the category of Support and Safety. In this context, care‐related pressure was perceived as an expression of concern, which in turn fostered feelings of support and safety within the therapeutic relationship (Tan et al. 2010).

#### 4.5.4 Impacts on Recovery

Informal coercion was identified as having adverse effects on adolescents' recovery experiences (*n* = 3). One subcategory, worsening of condition and symptoms, captured how rule‐based restrictions and rigid institutional routines were experienced as unhelpful or even obstructive to recovery. In particular, inhumane practices and a lack of flexibility were seen to hinder emotional and psychological progress, failing to support adolescents' subjective experiences of recovery (Rice et al. 2021). Furthermore, limitations on choice and participation were perceived to restrict the expression of symptoms, thereby complicating the recovery process (Tan et al. 2010).

#### 4.5.5 Impacts on Health Behaviour

Informal coercion was also found to influence adolescents' health behaviour (*n* = 1), particularly in relation to motivation and treatment engagement. Among the included studies, only one explicitly addressed this connection, suggesting that informal coercion may be associated with health‐promoting behaviours. In this context, external pressure was perceived to enhance adolescents' commitment to treatment and to encourage its continuation or maintenance (Moses 2011).

## 5 Discussion

This systematic review aimed to identify the forms of informal coercion experienced by adolescents in mental health care and to synthesise the findings available in existing literature. A total of 12 studies were included, from which seven distinct forms of informal coercion were identified (treatment pressure, referring to rules and routines, threat, (using) a disciplinary style, physical force, interpersonal leverage and deception). In addition, two novel forms of informal coercion emerged specifically from adolescents' own accounts (silencing and exclusion and manipulation). These forms were present across a range of mental health service settings, including both inpatient and outpatient care.

Another key aim of this review was to explore the consequences of informal coercion as experienced by adolescents. The findings suggest that such practices have consequences on adolescents' reactive coping strategies, emotional wellbeing, relational impacts on the care relationship, experienced impacts on recovery and health behaviour. Although the aim of this systematic review was not to compare positive and negative experiences, it is noteworthy that the consequences of informal coercion were predominantly described as negative across the included studies. In contrast, positive or ambivalent interpretations were considerably less common. This asymmetry highlights the potential of informal coercion to undermine adolescents' engagement in treatment and their recovery processes. While some adolescents perceived structure and pressure as protective, the majority described their experiences as coercive, which in turn diminished their sense of autonomy and trust. For mental health nursing, these findings underscore the importance of fostering therapeutic relationships that balance guidance with respect for autonomy and support adolescents' active participation in care.

Notably, none of the included studies explicitly focused on informal coercion as a primary research topic, highlighting a significant gap in the current evidence base. To the best of our knowledge, no dedicated empirical research has systematically explored adolescents' experiences of informal coercion in mental health care. More broadly, the limited number of studies addressing adolescents' perspectives in this context reflects a wider paucity of research concerning adolescents' views and experiences in mental health care (MacDonald et al. 2021). As a result, decisions regarding the organisation, content and quality of adolescent mental health care continue to be shaped predominantly by adult‐centric perspectives (Scherer and Reppucci 1988). This is problematic, as research has shown that adolescents' experiences of recovery (Moberg et al. 2023), mental health disorders (Twivy et al. 2023) and overall symptom profiles (Rice et al. 2019) differ from those of adults. Furthermore, studies have identified themes that are specific to adolescence, such as social identity, school‐related concerns and developmental challenges (Fusar‐Poli et al. 2024; Twivy et al. 2023), which further distinguish adolescents' experiences from adult populations.

These findings are consistent with those of the present review, which suggest that existing conceptualisations of informal coercion, based primarily on the perspectives of adult patients and professionals, may not fully capture the full breadth and complexity of informal coercion forms as experienced by adolescents in mental health care. This underscores the importance of incorporating adolescents' lived experiences into both the design and delivery of adolescent mental health care. Therefore, a deeper understanding of informal coercion and adolescents' lived experiences is essential to advance mental health nursing knowledge and the development of evidence‐based, developmentally sensitive and ethically grounded care practices.

Although not the primary focus of this review, the analysis revealed several underlying and contextual factors that may contribute to the use of informal coercion, offering important directions for future research. The care environment appeared to shape how informal coercion was manifested and experienced. Adolescents described various forms of informal coercion as instruments of power used to influence their thinking and behaviour, grounded in power asymmetries, an observation also noted by Hempeler et al. (2024). In some cases, informal coercion seemed to function as a pedagogical tool aimed at directing adolescents' behaviour or attitudes, raising important questions about the perceived legitimacy and ethical implications of such practices in mental health nursing.

These observations are supported by previous research on both formal and informal coercion, which has emphasised the context‐dependent nature of coercive practices in adolescent mental health care. Key influencing factors identified in earlier studies include the care culture of the unit and professionals' competencies (Moell et al. 2025), knowledge asymmetries between professionals and adolescents and broader social environments such as family dynamics (Nyttingnes et al. 2018). Therefore, future studies within mental health nursing should approach informal coercion from a perspective that more comprehensively considers adolescents' social context and how it shapes their experiences in mental health care.

Ultimately, informal coercion appears to be deeply embedded in adult‐led mental health care systems and reflects broader societal discourses on adolescents' rights to autonomy. These discourses often justify restrictions based on assumptions of developmental immaturity and the need for protection. Increasingly, however, there is recognition of the need for more nuanced and individualised assessments of adolescents' decision‐making capacity (Scherer and Reppucci 1988). For example, Knaappila et al. (2021) reflected on the relevance of the maturity gap theory (Dijkstra et al. 2015) in interpreting the observed decline in externalising behaviours preceding the recent rise in internalising symptoms among adolescents. The theory suggests that adolescents may experience a mismatch between their biological maturation marked by a growing desire for autonomy and the limited social autonomy afforded to them in contemporary Western societies. This discrepancy can lead to frustration, which some adolescents may express through delinquent acts or other forms of risk behaviour (Dijkstra et al. 2015; Knaappila et al. 2021). As they begin to perceive themselves as autonomous individuals, such behaviours often diminish, suggesting that increased autonomy may support more adaptive developmental outcomes (Barnes and Beaver 2010). In the context of mental health care, this perspective offers a useful lens for understanding how informal coercion may be experienced by adolescents. When perceived as undermining their developing autonomy, informal coercion may contribute to frustration, mistrust and a sense of disempowerment, potentially weakening therapeutic alliances and reducing engagement in care. These findings underscore the importance of developmentally sensitive approaches that actively support adolescents' autonomy and participation in decision‐making processes in mental health nursing.

This review has a few important limitations. First, the number of included studies was relatively small (*n* = 12), which limits the generalisability of the findings and reflects the overall scarcity of research explicitly addressing adolescents' experiences of informal coercion. Second, none of the studies focused on informal coercion as a primary research topic, which means that the identification of coercive practices relied on secondary interpretations of data, potentially affecting the depth and specificity of the findings. Third, the heterogeneity of study designs, settings and populations posed challenges for synthesis. The included studies varied in terms cultural context, and service structures, which may have influenced how informal coercion was perceived and experienced. In addition, while the review aimed to capture adolescents' lived experiences, many of the studies included perspectives mediated through adult researchers or clinicians, which may have shaped the framing and interpretation of adolescents' voices. Two of the included studies employed retrospective designs, which may have introduced positivity bias (Michalos 2014), and may affect the reliability of adolescents' accounts. Finally, the search strategy focused on terms related to coercion and mental health care, which may have excluded studies using alternative conceptual frameworks (e.g., relational ethics, behavioural management, or pedagogical approaches) that nonetheless describe coercive dynamics.

## 6 Conclusion

The findings reveal that informal coercion is not only present but multifaceted, encompassing both previously recognised and novel forms such as silencing and exclusion and manipulation. These practices were identified across diverse mental health care settings and were experienced as having consequences on emotions, care relations, health‐related behaviours and recovery. While some experiences were interpreted as protective or supportive, the majority were perceived as disempowering, distressing and undermining autonomy. Importantly, adolescents' responses to informal coercion included both adaptive and oppositional coping strategies, highlighting the complex interplay between agency, context and perceived legitimacy of care.

The review underscores a critical gap in the current evidence base: existing conceptualisations of informal coercion are largely derived from adult and professional perspectives and may fail to capture the relational, subtle and context‐dependent nature of coercion as experienced by adolescents. Incorporating adolescents' lived experiences into both the conceptual understanding of informal coercion and the practical design of adolescents' mental health care is thus essential. Such an approach would not only enhance the validity of informal coercion constructs but also support the development of more collaborative, developmentally attuned models of mental health nursing. Future research should aim to address these limitations by conducting dedicated empirical studies that centre adolescents' voices, employ participatory and youth‐informed methodologies and explore informal coercion across diverse cultural and service contexts.

## 7 Relevance for Clinical Practice

This review synthesises new findings about the forms of informal coercion as experienced by adolescents and may inform the development of mental health nursing that better acknowledges adolescents' experiences and the complex forms of informal coercion. This direction aligns with the mission of the WPA‐CAP and the World Health Organization, Regional Office for Europe's (2025) quality standards for youth mental health services, both of which emphasise adherence to the highest standards of clinical and ethical practice globally. Advancing research and practice requires collecting and analysing data on informal coercion as experienced and understood by adolescent patients themselves.

## Author Contributions

All authors listed meet the authorship criteria according to the latest guidelines of the International Committee of Medical Journal Editors, and all authors are in agreement with the manuscript. T.Ö. and T.L. contributed substantially to the conception of the work. T.Ö. acquired the data. T.Ö. and T.L. analysed the data. T.Ö. interpreted the data and drafted the manuscript. T.Ö. and T.L. critically reviewed the article for its important intellectual content.

## Funding

The authors have nothing to report.

## Disclosure

The authors have nothing to report.

## Ethics Statement

The authors have nothing to report.

## Conflicts of Interest

The authors declare no conflicts of interest.

## Supporting information


**Table S1:** inm70245‐sup‐0001‐TableS1.docx.

**Table S2:** inm70245‐sup‐0002‐TableS2.docx.

## Data Availability

The data that support the findings of this study are available from the corresponding author upon reasonable request.

## References

[inm70245-bib-0001] Allison, R. , and K. Flemming . 2019. “Mental Health Patients' Experiences of Softer Coercion and Its Effects on Their Interactions With Practitioners: A Qualitative Evidence Synthesis.” Journal of Advanced Nursing 75, no. 11: 2274–2284. 10.1111/jan.14035. 31012149

[inm70245-bib-0002] Andersson, U. , J. Fathollahi , and L. W. Gustin . 2020. “Nurses' Experiences of Informal Coercion on Adult Psychiatric Wards.” Nursing Ethics 27, no. 3: 741–753. 10.1177/0969733019884604. 31898470

[inm70245-bib-0003] Barnes, J. C. , and K. M. Beaver . 2010. “An Empirical Examination of Adolescence‐Limited Offending: A Direct Test of Moffitt's Maturity Gap Thesis.” Journal of Criminal Justice 38, no. 6: 1176–1185. 10.1016/j.jcrimjus.2010.09.006.

[inm70245-bib-0004] Beeri, S. , E. Baumberger , S. Zwakhalen , and S. Hahn . 2025. “Conceptualisation of Informal Coercion in Inpatient Psychiatry: A Scoping Review.” International Journal of Mental Health Nursing 34, no. 3: e70076. 10.1111/inm.70076. 40474445

[inm70245-bib-0005] Bjønness, S. , T. Grønnestad , and M. Storm . 2020. “I'm Not a Diagnosis: Adolescents' Perspectives on User Participation and Shared Decision‐Making in Mental Healthcare.” Scandinavian Journal of Child and Adolescent Psychiatry and Psychology 8, no. 1: 139–148. 10.21307/sjcapp-2020-014. 33564630 PMC7863730

[inm70245-bib-0006] Collet, O. A. , M. Orri , C. Galéra , et al. 2025. “Children's Mental Health Symptoms Over Three Decades (1993–2022): A Comparison of Population‐Based Cross‐Sectional Samples.” European Child & Adolescent Psychiatry 35: 285–293. 10.1007/s00787-025-02854-y. 40982044 PMC12916540

[inm70245-bib-0007] Coyne, I. , N. McNamara , M. Healy , C. Gower , M. Sarkar , and F. McNicholas . 2015. “Adolescents' and Parents' Views of Child and Adolescent Mental Health Services (CAMHS) in Ireland.” Journal of Psychiatric and Mental Health Nursing 22, no. 8: 561–569. 10.1111/jpm.12215. 25977175

[inm70245-bib-0008] Dijkstra, J. K. , T. Kretschmer , K. Pattiselanno , et al. 2015. “Explaining Adolescents' Delinquency and Substance Use: A Test of the Maturity Gap: The SNARE Study.” Journal of Research in Crime and Delinquency 52, no. 5: 747–767. 10.1177/0022427815582249.

[inm70245-bib-0009] Elmer, T. , F. Rabenschlag , D. Schori , et al. 2018. “Informal Coercion as a Neglected Form of Communication in Psychiatric Settings in Germany and Switzerland.” Psychiatry Research 262: 400–406. 10.1016/j.psychres.2017.09.014. 28958458

[inm70245-bib-0010] Elo, S. , and H. Kyngäs . 2008. “The Qualitative Content Analysis Process.” Journal of Advanced Nursing 62, no. 1: 107–115. 10.1111/j.1365-2648.2007.04569.x. 18352969

[inm70245-bib-0011] Engström, I. , K. Engström , and T. Sellin . 2020. “Adolescents' Experiences of the Staff's Different Interaction Styles in Coercive Youth Care in Sweden: A Qualitative Study.” Issues in Mental Health Nursing 41, no. 11: 1027–1037. 10.1080/01612840.2020.1757794. 32585115

[inm70245-bib-0012] Fusar‐Poli, P. , A. Estradé , C. M. Esposito , et al. 2024. “The Lived Experience of Mental Disorders in Adolescents: A Bottom‐Up Review Co‐Designed, Co‐Conducted and Co‐Written by Experts by Experience and Academics.” World Psychiatry 23, no. 2: 191–208. 10.1002/wps.21189. 38727047 PMC11083893

[inm70245-bib-0013] Gonzalez‐Urbina, A. 2022. “Psychiatric Power and Adultcentrism. Children's and Adolescent's Experiences of Coercion in the Psychiatric Ward.” Rassegna Italiana di Sociologia 4: 879–903. 10.1423/106248.

[inm70245-bib-0014] Hempeler, C. , E. Braun , S. Potthoff , J. Gather , and M. Scholten . 2024. “When Treatment Pressures Become Coercive: A Context‐Sensitive Model of Informal Coercion in Mental Healthcare.” American Journal of Bioethics 24, no. 12: 74–86. 10.1080/15265161.2023.2232754. 37506325

[inm70245-bib-0015] Hong, Q. N. , P. Pluye , S. Fàbregues , et al. 2019. “Improving the Content Validity of the Mixed Methods Appraisal Tool: A Modified e‐Delphi Study.” Journal of Clinical Epidemiology 111: 49–59. 10.1016/j.jclinepi.2019.03.008. 30905698

[inm70245-bib-0016] Hotzy, F. , and M. Jaeger . 2016. “Clinical Relevance of Informal Coercion in Psychiatric Treatment—A Systematic Review.” Frontiers in Psychiatry 7: 197. 10.3389/fpsyt.2016.00197. 28018248 PMC5149520

[inm70245-bib-0017] Høyer, G. , L. Kjellin , M. Engberg , et al. 2002. “Paternalism and Autonomy: A Presentation of a Nordic Study on the Use of Coercion in the Mental Health Care System.” International Journal of Law and Psychiatry 25, no. 2: 93–108. 12071105 10.1016/s0160-2527(01)00108-x

[inm70245-bib-0018] Hsieh, H.‐F. , and S. E. Shannon . 2005. “Three Approaches to Qualitative Content Analysis.” Qualitative Health Research 15, no. 9: 1277–1288. 10.1177/1049732305276687. 16204405

[inm70245-bib-0019] Jaeger, M. , and W. Rossler . 2010. “Enhancement of Outpatient Treatment Adherence: Patients' Perceptions of Coercion, Fairness and Effectiveness.” Psychiatry Research 180, no. 1: 48–53. 10.1016/j.psychres.2009.09.011. 20493550

[inm70245-bib-0020] Jones, N. , B. K. Gius , M. Shields , S. Collings , C. Rosen , and M. Munson . 2021. “Investigating the Impact of Involuntary Psychiatric Hospitalization on Youth and Young Adult Trust and Help‐Seeking in Pathways to Care.” Social Psychiatry and Psychiatric Epidemiology 56, no. 11: 2017–2027. 10.1007/s00127-021-02048-2. 33751175 PMC10105343

[inm70245-bib-0021] Knaappila, N. , M. Marttunen , S. Fröjd , and R. Kaltiala . 2021. “Changes Over Time in Mental Health Symptoms Among Adolescents in Tampere, Finland.” Scandinavian Journal of Child and Adolescent Psychiatry and Psychology 9, no. 1: 96–104. 10.21307/sjcapp-2021-011. 34079771 PMC8132727

[inm70245-bib-0022] LeFrançois, B. 2008. ““It's Like Mental Torture”: Participation and Mental Health Services.” International Journal of Children's Rights 16, no. 2: 211–227. 10.1163/157181808X301809.

[inm70245-bib-0023] Lidz, C. W. , E. P. Mulvey , S. K. Hoge , et al. 1998. “Factual Sources of Psychiatric Patients' Perceptions of Coercion in the Hospital Admission Process.” American Journal of Psychiatry 155, no. 9: 1254–1260. 10.1176/ajp.155.9.1254. 9734551

[inm70245-bib-0052] Lorem, G. F. , M. H. Hem , and B. Molewijk . 2015. “Good Coercion: Patients’ Moral Evaluation of Coercion in Mental Health Care.” International Journal of Mental Health Nursing 24, no. 3: 231–240. 10.1111/inm.12106. 25394674

[inm70245-bib-0024] Ludot‐Grégoire, M. , V. David , E. Carretier , J. Lachal , M. R. Moro , and C. Blanchet . 2022. “Subjective Experience of Antidepressant Prescription Among Adolescents With Anorexia Nervosa.” Frontiers in Psychiatry 13: 770 903. 10.3389/fpsyt.2022.770903. PMC9013857 35444576

[inm70245-bib-0025] Lützén, K. 1998. “Subtle Coercion in Psychiatric Practice.” Journal of Psychiatric and Mental Health Nursing 5, no. 2: 101–107. 10.1046/j.1365-2850.1998.00104.x. 9661411

[inm70245-bib-0026] MacDonald, K. , M. Ferrari , N. Fainman‐Adelman , and S. N. Iyer . 2021. “Experiences of Pathways to Mental Health Services for Young People and Their Carers: A Qualitative Meta‐Synthesis Review.” Social Psychiatry and Psychiatric Epidemiology 56, no. 3: 339–361. 10.1007/s00127-020-01976-9. 33206200

[inm70245-bib-0027] Michalos, A. C. , ed. 2014. Encyclopedia of Quality of Life and Well‐Being Research. Springer. 10.1007/978-94-007-0753-5.

[inm70245-bib-0028] Moberg, J. , L. Skogens , and U. Schön . 2023. “Review: Young People's Recovery Processes From Mental Health Problems—A Scoping Review.” Child and Adolescent Mental Health 28, no. 3: 393–407. 10.1111/camh.12594. 35960215

[inm70245-bib-0029] Moell, A. , M. S. Lyle , A. Rozental , and N. Långström . 2024. “Rates and Risk Factors of Coercive Measure Use in Inpatient Child and Adolescent Mental Health Services: A Systematic Review and Narrative Synthesis.” Lancet Psychiatry 11, no. 10: 839–852. 10.1016/S2215-0366(24)00204-9. 39121879

[inm70245-bib-0030] Moell, A. , A. Rozental , S. Buchmayer , R. Kaltiala , and N. Långström . 2025. “Perceived Determinants of the Use of Coercion in Inpatient Child and Adolescent Psychiatry: A Qualitative Interview Study With Staff.” BMC Psychiatry 25, no. 1: 246. 10.1186/s12888-025-06690-x. 40091017 PMC11912791

[inm70245-bib-0031] Moses, T. 2011. “Adolescents' Commitment to Continuing Psychotropic Medication: A Preliminary Investigation of Considerations, Contradictions, and Correlates.” Child Psychiatry & Human Development 42, no. 1: 93–117. 10.1007/s10578-010-0209-y. 20953829

[inm70245-bib-0032] Neale, M. S. , and R. A. Rosenheck . 2000. “Therapeutic Limit Setting in an Assertive Community Treatment Program.” Psychiatric Services 51, no. 4: 499–505. 10.1176/appi.ps.51.4.499. 10737826

[inm70245-bib-0033] Nyttingnes, O. , T. Ruud , R. Norvoll , J. Rugkåsa , and K. Hanssen‐Bauer . 2018. “A Cross‐Sectional Study of Experienced Coercion in Adolescent Mental Health Inpatients.” BMC Health Services Research 18, no. 1: 389. 10.1186/s12913-018-3208-5. 29848338 PMC5977498

[inm70245-bib-0034] Offord, A. , H. Turner , and M. Cooper . 2006. “Adolescent Inpatient Treatment for Anorexia Nervosa: A Qualitative Study Exploring Young Adults' Retrospective Views of Treatment and Discharge.” European Eating Disorders Review 14, no. 6: 377–387. 10.1002/erv.687.

[inm70245-bib-0051] Page, M. J. , J. E. McKenzie , P. M. Bossuyt , et al. 2021. “The PRISMA 2020 Statement: An Updated Guideline for Reporting Systematic Reviews.” BMJ 372: n71. 33782057 10.1136/bmj.n71 PMC8005924

[inm70245-bib-0035] Pelto‐Piri, V. , L. Kjellin , U. Hylén , E. Valenti , and S. Priebe . 2019. “Different Forms of Informal Coercion in Psychiatry: A Qualitative Study.” BMC Research Notes 12, no. 1: 787. 10.1186/s13104-019-4823-x. 31791408 PMC6889621

[inm70245-bib-0036] Persson, S. , C. Hagquist , and D. Michelson . 2017. “Young Voices in Mental Health Care: Exploring Children's and Adolescents' Service Experiences and Preferences.” Clinical Child Psychology and Psychiatry 22, no. 1: 140–151. 10.1177/1359104516656722. 27368712

[inm70245-bib-0037] Potthoff, S. , J. Gather , C. Hempeler , A. Gieselmann , and M. Scholten . 2022. ““Voluntary in Quotation Marks”: A Conceptual Model of Psychological Pressure in Mental Healthcare Based on a Grounded Theory Analysis of Interviews With Service Users.” BMC Psychiatry 22, no. 1: 186. 10.1186/s12888-022-03810-9. 35296288 PMC8928679

[inm70245-bib-0038] Rice, F. , L. Riglin , T. Lomax , et al. 2019. “Adolescent and Adult Differences in Major Depression Symptom Profiles.” Journal of Affective Disorders 243: 175–181. 10.1016/j.jad.2018.09.015. 30243197

[inm70245-bib-0039] Rice, J. L. , T. X. Tan , and Y. Li . 2021. “In Their Voices: Experiences of Adolescents During Involuntary Psychiatric Hospitalization.” Children and Youth Services Review 126: 106045. 10.1016/j.childyouth.2021.106045.

[inm70245-bib-0040] Rugkåsa, J. , K. Canvin , J. Sinclair , A. Sulman , and T. Burns . 2014. “Trust, Deals and Authority: Community Mental Health Professionals' Experiences of Influencing Reluctant Patients.” Community Mental Health Journal 50, no. 8: 886–895. 10.1007/s10597-014-9720-0. 24664366

[inm70245-bib-0041] Scherer, D. G. , and N. D. Reppucci . 1988. “Adolescents' Capacities to Provide Voluntary Informed Consent: The Effects of Parental Influence and Medical Dilemmas.” Law and Human Behavior 12, no. 2: 123–141. 10.1007/BF01073121. 11653858

[inm70245-bib-0042] Solmi, M. , J. Radua , M. Olivola , et al. 2022. “Age at Onset of Mental Disorders Worldwide: Large‐Scale Meta‐Analysis of 192 Epidemiological Studies.” Molecular Psychiatry 27, no. 1: 281–295. 10.1038/s41380-021-01161-7. 34079068 PMC8960395

[inm70245-bib-0043] Szmukler, G. , and P. S. Appelbaum . 2008. “Treatment Pressures, Leverage, Coercion, and Compulsion in Mental Health Care.” Journal of Mental Health 17, no. 3: 233–244. 10.1080/09638230802052203.

[inm70245-bib-0044] Tan, J. O. A. , A. Stewart , R. Fitzpatrick , and T. Hope . 2010. “Attitudes of Patients With Anorexia Nervosa to Compulsory Treatment and Coercion.” International Journal of Law and Psychiatry 33, no. 1: 13–19. 10.1016/j.ijlp.2009.10.003. 19926134 PMC2808473

[inm70245-bib-0045] Twivy, E. , M. Kirkham , and M. Cooper . 2023. “The Lived Experience of Adolescent Depression: A Systematic Review and Meta‐Aggregation.” Clinical Psychology & Psychotherapy 30, no. 4: 754–766. 10.1002/cpp.2834. 36700415

[inm70245-bib-0046] Valenti, E. , C. Banks , A. Calcedo‐Barba , et al. 2015. “Informal Coercion in Psychiatry: A Focus Group Study of Attitudes and Experiences of Mental Health Professionals in Ten Countries.” Social Psychiatry and Psychiatric Epidemiology 50, no. 8: 1297–1308. 10.1007/s00127-015-1032-3. 25720809 PMC7521205

[inm70245-bib-0047] Walker, S. , P. Barnett , R. Srinivasan , E. Abrol , and S. Johnson . 2021. “Clinical and Social Factors Associated With Involuntary Psychiatric Hospitalisation in Children and Adolescents: A Systematic Review, Meta‐Analysis, and Narrative Synthesis.” Lancet Child & Adolescent Health 5, no. 7: 501–512. 10.1016/S2352-4642(21)00089-4. 33930330 PMC8205858

[inm70245-bib-0048] World Health Organization, Regional Office for Europe . 2025. “Quality Standards for Child and Youth Mental Health Services: For Use in Specialized Community or Outpatient Care Across the WHO European Region.” World Health Organization, Regional Office for Europe. https://apps.who.int/iris/handle/10665/.

[inm70245-bib-0049] World Psychiatric Association . n.d. “Child and Adolescent Psychiatry Section.” World Psychiatric Association. Accessed November 4, 2025. https://www.wpanet.org/section‐page/childand‐adolescent‐psychiatry/.

[inm70245-bib-0050] Yeeles, K. 2016. Informal Coercion: Current Evidence (Vol. 1). Oxford University Press. 10.1093/med/9780198788065.003.0006.

