# Supplementary material for: Informal Coercion Experienced by Adolescents in Mental Health Care—A Systematic Review
Source: Int J Ment Health Nurs. 2026 Mar 12;35(2):e70245. doi: 10.1111/inm.70245 (PMC12982914; doi:10.1111/inm.70245)
Supplement: Supplementary file 1 — Table S1: inm70245‐sup‐0001‐TableS1.docx. [file INM-35-0-s001.docx]

| SUPPLEMENTARY TABLE 1 Search Strategy Terms | | |
| --- | --- | --- |
| APAPsycInfo | Population (P) | adolescent* OR teen* OR young* OR youth* OR "young people" OR youngster* OR junior* OR juven* OR minor*) |
|  | Intervention (I) | DE "Coercion" OR coercion OR coercive OR coerced)  AND  (DE "Persuasive Communication" OR DE "Threat" OR DE "Interpersonal Control" OR DE "Abuse of Power" OR DE "Social Influences" OR DE "Paternalism" OR DE "Autonomy" OR DE "Freedom" OR DE "Human Rights" OR DE "Persuasion" OR DE "Persuasive Communication" OR DE "Deception" OR DE "Interpersonal Influences" OR DE "Cheating" OR informal OR subtle* OR soft* OR hidden OR mild* OR modest OR covert OR leverage OR threat* OR threatening OR control* OR pressure* OR pressuring OR persuasion OR persuade OR persuasive OR power OR "power over" OR punitive OR influence OR influencing OR intimidation OR intimidate OR intimidating OR blackmail* OR negotiation OR negotiate OR negotiating OR inducement OR deception* OR restriction* OR restrict* OR restrictive OR perception* OR perceived OR perceive OR perceiving OR "treatment pressure*" OR "treatment relationship*" OR "therapeutic relationship*" OR "care relationship*" OR "blanket restriction*" OR "blanket rule*" OR "house restriction*" OR "house rule*" OR paternalism OR paternalistic OR autonomy OR ethics* OR voluntariness OR denial OR deny OR free* OR cheat*) |
|  | Setting | DE "Mental Health Services" OR DE "Community Mental Health Services" OR DE "Psychiatric Clinics" OR DE "Community Mental Health Centers" OR DE "Psychiatric Units" OR DE "Psychiatric Hospitals" OR DE "Mental Disorders" OR DE "Affective Disorders" OR DE "Anxiety Disorders" OR DE "Behavior Disorders" OR DE "Bipolar Disorder" OR DE "Borderline States" OR DE "Chronic Mental Illness" OR DE "Dissociative Disorders" OR DE "Eating Disorders" OR DE "Gender Dysphoria" OR DE "Mental Disorders due to General Medical Conditions" OR DE "Neurocognitive Disorders" OR DE "Neurodevelopmental Disorders" OR DE "Neurosis" OR DE "Obsessive Compulsive Disorder" OR DE "Paraphilias" OR DE "Personality Disorders" OR DE "Psychosis" OR DE "Serious Mental Illness" OR DE "Sleep Wake Disorders" OR DE "Somatoform Disorders" OR DE "Stress and Trauma Related Disorders" OR "psychiatric care" OR "psychiatric ward*" OR "mental health service*" OR psychiatry OR psychiatric OR "psychiatric treatment*" OR "psychiatric hospital*" OR "mental health clinic*" OR "mental health care" OR "mental healthcare" OR "psychiatric setting*" OR "psychiatric care" OR "mental health clinic*" OR mental OR mentally OR "bipolar disorder*" OR depression OR depressive OR schizophrenia OR schizophrenic OR psychotic OR psychosis OR psychoses OR "personality disorder*") |

| SUPPLEMENTARY TABLE 1 Continued | | |
| --- | --- | --- |
| CINAHL | Population (P) | MH "Adolescence+" OR teen* OR adolescent* OR young* OR youth* OR "young people" OR youngster* OR junior* OR juven* OR minor* |
|  | Intervention (I) | MH "Coercion" OR coercion OR coercive OR coerced  AND  MH "Paternalism" OR MH "Autonomy" OR MH "Patient Autonomy" OR MH "Relational Autonomy" OR MH "Control (Psychology)+" OR MH "Pressure (Physiology)+" OR MH "Persuasive Communication" OR MH "Power+" OR MH "Negotiation" OR MH "Deception" OR informal OR subtle* OR soft* OR hidden OR mild* OR modest OR covert OR leverage OR threat* OR threatening OR control* OR pressure* OR pressuring OR persuasion OR persuade OR persuasive OR power OR "power over" OR punitive OR influence OR influencing OR intimidation OR intimidate OR intimidating OR blackmail* OR negotiation OR negotiate OR negotiating OR inducement OR restriction* OR restrict* OR restrictive OR perception* OR perceived OR perceive OR perceiving OR "treatment pressure*" OR "treatment relationship*" OR "therapeutic relationship*" OR "care relationship*" OR "blanket restriction*" OR "blanket rule*" OR "house restriction*" OR "house rule*" OR paternalism OR paternalistic OR autonomy OR ethics* OR voluntariness OR denial OR deny OR free* OR cheat* |
|  | Setting | MH "Mental Disorders" OR MH "Hospitals, Psychiatric" OR MH "Psychiatric Units" OR MH "Psychiatric Care" OR MH "Psychiatric Service" OR "psychiatric care" OR "psychiatric treatment" OR "psychiatric ward*" OR "mental health service*" OR psychiatry OR psychiatric OR "psychiatric hospital*" OR "psychiatric treatment*" OR "mental health clinic*" OR "mental health care" OR "mental healthcare" OR "psychiatric setting*" OR "psychiatric treatment*" OR "psychiatric care" OR "mental health clinic*" OR mental OR mentally OR "bipolar disorder*" OR depression OR depressive OR schizophrenia OR schizophrenic OR psychotic OR psychosis OR psychoses OR "personality disorder*") |

| SUPPLEMENTARY TABLE 1 Continued | |
| --- | --- |
| Cochrane Library | ID Search Hits  #1 MeSH descriptor: [Coercion] explode all trees 128  #2 (coercion OR coercive OR coerced) 672  #3 #1 OR #2 672  #4 MeSH descriptor: [Personal Autonomy] explode all trees 351  #5 MeSH descriptor: [Paternalism] this term only 21  #6 MeSH descriptor: [Deception] this term only 213  #7 MeSH descriptor: [Negotiating] 3 tree(s) exploded 171  #8 MeSH descriptor: [Power, Psychological] explode all trees 551  #9 MeSH descriptor: [Persuasive Communication] this term only 415  #10 (informal OR subtle* OR soft* OR hidden OR mild* OR modest OR covert OR leverage OR threat* OR threatening OR control* OR pressure* OR pressuring OR persuasion OR persuade OR persuasive OR power OR power NEXT over OR punitive OR influence OR influencing OR intimidation OR intimidate OR intimidating OR blackmail* OR negotiation OR negotiate OR negotiating OR inducement OR deception* OR restriction* OR restrict* OR restrictive OR perception* OR perceived OR perceive OR perceiving OR treatment NEXT pressure* OR pressuring OR treatment NEXT relationship* OR therapeutic NEXT relationship* OR care NEXT relationship* OR blanket NEXT restriction* OR blanket NEXT rule* OR house NEXT restriction* OR house NEXT rule* OR paternalism OR paternalistic OR autonomy OR ethics* OR voluntariness OR denial OR deny OR free* OR cheat*) 2255093  #11 #4 OR #5 OR #6 OR #7 OR #8 OR #9 OR #10 2255093  #12 #3 AND #11 672  #13 MeSH descriptor: [Adolescent] this term only 139606  #14 (adolescent* OR teen* OR young* OR youth* OR "young people" OR youngster* OR junior* OR juven* OR minor*) 331111  #15 #13 OR #14 331111  #16 #12 AND #15 299  #17 MeSH descriptor: [Psychiatric Department, Hospital] this term only 136  #18 MeSH descriptor: [Mental Disorders] explode all trees 110307  #19 MeSH descriptor: [Mental Health Services] 1 tree(s) exploded 9690  #20 (psychiatric NEXT care OR psychiatric NEXT ward* OR mental NEXT health NEXT service* OR psychiatry OR psychiatric OR psychiatric NEXT hospital* OR mental NEXT health NEXT clinic* OR mental NEXT health NEXT care OR mental NEXT healthcare OR psychiatric NEXT setting* OR psychiatric NEXT treatment* OR psychiatric NEXT care OR mental NEXT health NEXT clinic* OR mental OR mentally OR bipolar NEXT disorder* OR anxiety OR depression OR depressive OR schizophrenia OR schizophrenic OR psychotic OR psychosis OR psychoses OR personality NEXT disorder*) 263100  #21 #17 OR #18 OR #19 OR #20 308401  #22 #16 AND #21 162 |

| SUPPLEMENTARY TABLE 1 Continued | | |
| --- | --- | --- |
| EMBASE | Population (P) | 'adolescent'/exp OR teen* OR adolescent* OR young* OR youth* OR "young people" OR youngster* OR junior* OR juven* OR minor* |
|  | Intervention (I) | 'coercion'/exp OR coercion OR coercive OR coerced  AND  'threat'/exp OR 'persuasive communication'/exp OR 'abuse of power'/exp OR 'intimidation'/exp OR 'interpersonal communication'/exp OR 'negotiation'/exp OR 'paternalism'/exp OR 'patient autonomy'/exp OR 'personal autonomy'/exp OR 'persuasive communication'/exp OR informal OR subtle* OR soft* OR hidden OR mild* OR modest OR covert OR leverage OR threat* OR threatening OR control* OR pressure* OR pressuring OR persuasion OR persuade OR persuasive OR power OR "power over" OR punitive OR influence OR influencing OR intimidation OR intimidate OR intimidating OR blackmail* OR negotiation OR negotiate OR negotiating OR inducement OR deception* OR restriction* OR restrict* OR restrictive OR perception* OR perceived OR perceive OR perceiving OR "treatment pressure*" OR "treatment relationship*" OR "therapeutic relationship*" OR "care relationship*" OR "blanket restriction*" OR "blanket rule*" OR "house restriction*" OR "house rule*" OR paternalism OR paternalistic OR autonomy OR ethics* OR voluntariness OR denial OR deny OR free* OR cheat* |
|  | Setting | 'mental disease'/exp OR 'psychiatric treatment'/exp OR 'psychiatric department'/exp OR 'mental hospital'/exp OR 'mental health care'/exp OR "psychiatric care" OR "psychiatric ward*" OR "mental health service*" OR psychiatry OR psychiatric OR "psychiatric hospital*" OR "psychiatric treatment*" OR "mental health clinic*" OR "mental health care" OR "mental healthcare" OR "psychiatric setting*" OR "psychiatric care" OR "mental health clinic*" OR mental OR mentally OR "bipolar disorder*" OR depression OR depressive OR anxiety OR schizophrenia OR schizophrenic OR psychotic OR psychosis OR psychoses OR "personality disorder*" |

| SUPPLEMENTARY TABLE 1 Continued | | |
| --- | --- | --- |
| PubMed | Population (P) | "Adolescent"[Mesh] OR teen* OR adolescent* OR young* OR youth* OR "young people" OR youngster* OR junior* OR juven* OR minor* |
|  | Intervention (I) | "Coercion"[Mesh] OR coercion OR coercive OR coerced  AND  "Paternalism"[Mesh] OR "Personal Autonomy"[Mesh] OR "Relational Autonomy"[Mesh] OR "Behavior Control"[Mesh] "perception"[Mesh:NoExp] OR "social control, informal"[MeSH] OR "Persuasive Communication"[MeSH] OR "negotiating/methods"[MeSH:noexp] OR "negotiating/psychology"[MeSH:noexp] OR "Negotiating"[MeSH:noexp] OR "Deception"[MeSH] OR "power, psychological"[MeSH] informal OR subtle* OR soft* OR hidden OR mild* OR modest OR covert OR leverage OR threat* OR threatening OR control* OR pressure* OR pressuring OR persuasion OR persuade OR persuasive OR power OR "power over" OR punitive OR influence OR influencing OR intimidation OR intimidate OR intimidating OR blackmail* OR negotiation OR negotiate OR negotiating OR inducement OR deception* OR restriction* OR restrict* OR restrictive OR perception* OR perceived OR perceive OR perceiving OR "treatment pressure*" OR "treatment relationship*" OR "therapeutic relationship*" OR "care relationship*" OR "blanket restriction*" OR "blanket rule*" OR "house restriction*" OR "house rule*" OR paternalism OR paternalistic OR autonomy OR ethics* OR voluntariness OR denial OR deny OR free* OR cheat* |
|  | Setting | "Mental Health Services"[Mesh] OR "Psychiatric Nursing"[Mesh] OR "Mental Health Services"[Mesh] OR "Community Psychiatry"[Mesh] OR "Preventive Psychiatry"[Mesh] OR "Community Mental Health Centers"[Mesh] OR "Adolescent Psychiatry"[Mesh] OR "Psychiatry"[Mesh] OR "Psychiatric Department, Hospital"[Mesh] OR "Community Psychiatry"[Mesh] OR "Hospitals, Psychiatric"[Mesh] OR "Mentally Ill Persons"[Mesh] OR "Mental Disorders"[Mesh] OR "psychiatric care" OR "psychiatric ward*" OR "mental health service*" OR psychiatry OR psychiatric OR "psychiatric hospital*" OR "mental health clinic*" OR "mental health care" OR "mental healthcare" OR "psychiatric setting*" OR "psychiatric care" OR "psychiatric treatment*" OR "mental health clinic*" OR mental OR mentally OR "bipolar disorder*" OR depression OR depressive OR schizophrenia OR schizophrenic OR psychotic OR psychosis OR psychoses OR "personality disorder*" |

| SUPPLEMENTARY TABLE 1 Continued | | |
| --- | --- | --- |
| SCOPUS/  Web of Science | Population (P) | teen* OR adolescent* OR young* OR youth* OR "young people" OR youngster* OR junior* OR juven* OR minor*) |
|  | Intervention (I) | coercion OR coercive OR coerced)  AND  (informal OR subtle* OR soft* OR hidden OR mild* OR modest OR covert OR leverage OR threat* OR threatening OR control* OR pressure* OR pressuring OR persuasion OR persuade OR persuasive OR power OR "power over" OR punitive OR influence OR influencing OR intimidation OR intimidate OR intimidating OR blackmail* OR negotiation OR negotiate OR negotiating OR inducement OR deception* OR restriction* OR restrict* OR restrictive OR perception* OR perceived OR perceive OR perceiving OR "treatment pressure*" OR "treatment relationship*" OR "therapeutic relationship*" OR "care relationship*" OR "blanket restriction*" OR "blanket rule*" OR "house restriction*" OR "house rule*" OR paternalism OR paternalistic OR autonomy OR ethics* OR voluntariness OR denial OR deny OR free* OR cheat* |
|  | Setting | ("psychiatric care" OR "psychiatric ward*" OR "mental health service*" OR psychiatry OR psychiatric OR "psychiatric hospital*" OR "mental health clinic*" OR "mental health care" OR "mental healthcare" OR "psychiatric setting*" OR "psychiatric treatment*" OR "psychiatric care" OR "mental health clinic*" OR mental OR mentally OR "bipolar disorder*" OR depression OR depressive OR anxiety OR schizophrenia OR schizophrenic OR psychotic OR psychosis OR psychoses OR "personality disorder*") |
