# Supplementary material for: Informal Coercion Experienced by Adolescents in Mental Health Care—A Systematic Review
Source: Int J Ment Health Nurs. 2026 Mar 12;35(2):e70245. doi: 10.1111/inm.70245 (PMC12982914; doi:10.1111/inm.70245)
Supplement: Supplementary file 2 — Table S2: inm70245‐sup‐0002‐TableS2.docx. [file INM-35-0-s002.docx]

SUPPLEMENTARY TABLE 2 Forms of informal coercion identified in previous literature

| Informal coercion | | |
| --- | --- | --- |
| **Forms (key concepts/variables)** | **Operational definitions** | **References** |
| Persuasion | Appealing to reason (but often also to emotions), the professional is concerned about the situation and keen to try to help and avert another damaging relapse. The discussion with the patient revolves around an arguably realistic appraisal of the benefits and risks of treatment. There is respect for the patient’s arguments and the treatment is discussed in the context of their value system, sometimes more as a negotiation. There is a verbal effort to get the person to sign into the hospital, relying on reason, the subject’s desire to please the persuader, and other motives that do not involve modifying the subject’s environment. | (Lidz et al. 1998; Pelto-Piri et al. 2019; Szmukler & Appelbaum 2008; Valenti et al. 2015) |
| Interpersonal leverage | Interpersonal leverage (or ‘‘interpersonal pressure’’) may be exercised through the emotional dependency that the patient might have on the professional. The patient may wish to please someone who has been helpful or react to signs of disappointment in the clinician when a treatment suggestion is rejected. The clinician tries to appeal to the patient. | (Pelto-Piri et al. 2019; Szmukler & Appelbaum 2008; Valenti et al. 2015) |
| Inducement | A negotiation-like situation in which a professional offers something to a patient in exchange for the patient’s action, such as the patient receiving some benefit that is important to them (coffee, an extra cigarette, a walk), if the patient is well enough, completes some treatment or, for example, accepts a course of medication. Whether or not the clinician means to imply that the patient needs to take treatment to gain his assistance is left unclear, but it remains the patient’s assumption. A conditional statement in which the potential patient is offered something positive in exchange for agreeing to admission. | (Lidz et al. 1998; Pelto-Piri et al. 2019; Szmukler & Appelbaum 2008; Valenti et al. 2015) |
| Threat | Inducement and threats are often considered together. The doctor explains that if the patient becomes increasingly irritable at the appointment or refuses treatment, the appointment may have to be cancelled and that he or she has a duty to inform social services. The professional, for example, points out that the patient may be entitled to a higher level of disability benefit, but if the application is rejected, the professional will not help with the appeal process if the patient does not accept the treatment ordered. Some can refer to form of care, such as “You can enter the hospital voluntarily, or we will have to commit you,” a conditional statement in which the potential patient is told that the threatener will do something negative if the potential patient does not agree to admission. | (Lidz et al. 1998; Neale & Rosenheck 2000; Pelto-Piri et al. 2019; Szmukler & Appelbaum 2008; Valenti et al. 2015) |
| Influence over/influencing behavior | Building trusting relationships to attempt to influence patients to achieve treatment aims, negotiating agreements focuses on “striking deals’’ or agreeing contracts. When deals failed or professionals were worried about the patient’s condition, more assertive methods (assertive authority) were used for ensuring medication treatment. | (Pelto-Piri et al. 2019; Rugkåsa et al. 2014) |
| (Using) a disciplinary style | “Like not saving any food if the patient was late for dinner or not allowing them to eat in the dining room when smelling bad” | (Pelto-Piri et al. 2019) |
| Referring to rules and routines | Verbally restricting or imposing by referring to rules and routines, such as “You can’t drink coffee whenever you want. You can’t smoke whenever you want. You can’t wear the clothes that you want to wear.” | (Pelto-Piri et al. 2019) |
| Treatment pressure | Refers to communicative strategies used to influence the decision-making of service users and improve their adherence to recommended treatment or social rules; refers to interventions aimed at inducing reluctant patients to accept treatment. | (Lorem et al. 2015; Potthoff et al. 2022) |
| Deception | Cheating (e.g., giving medicine without patient being aware of it). Also, lies or deliberate deceit of any sort. | (Lidz et al. 1998; Pelto-Piri et al. 2019) |
| Blackmail | The use of interpersonal leverage and inducements are at risk of turning into blackmail in some situations, e.g., in suicide threat situations. “I’d be really sad if you took your own life.” Using the therapeutic situation. | (Pelto-Piri et al. 2019) |
| Show of force | Show of force—an act that demonstrates the availability of force if it is needed (such as calling the police or hospital security). *Similarities with threat.* | (Lidz et al. 1998) |
| Physical force | An act involving the laying on of hands to accomplish something against the expressed choice of the potential patient. *Formal coercion excluded.* | (Lidz et al. 1998) |
| Giving orders | A statement related to admission in which someone stated that the patient had to do something; *this is distinguishable from a threat by the lack of a conditional result.* | (Lidz et al. 1998) |
